# The development of PIPA: an integrated and automated pipeline for genome-wide protein function annotation

**DOI:** 10.1186/1471-2105-9-52

**Published:** 2008-01-25

**Authors:** Chenggang Yu, Nela Zavaljevski, Valmik Desai, Seth Johnson, Fred J Stevens, Jaques Reifman

**Affiliations:** 1 Biotechnology HPC Software Applications Institute, Telemedicine and Advanced Technology Research Center, US Army Medical Research and Materiel Command, Ft. Detrick, MD, USA; 2 George Mason University, Manassas, VA, USA; 3 Biosciences Division, Argonne National Laboratory, Argonne, IL, USA

## Abstract

**Background:**

Automated protein function prediction methods are needed to keep pace with high-throughput sequencing. With the existence of many programs and databases for inferring different protein functions, a pipeline that properly integrates these resources will benefit from the advantages of each method. However, integrated systems usually do not provide mechanisms to generate customized databases to predict particular protein functions. Here, we describe a tool termed PIPA (Pipeline for Protein Annotation) that has these capabilities.

**Results:**

PIPA annotates protein functions by combining the results of multiple programs and databases, such as InterPro and the Conserved Domains Database, into common Gene Ontology (GO) terms. The major algorithms implemented in PIPA are: (1) a profile database generation algorithm, which generates customized profile databases to predict particular protein functions, (2) an automated ontology mapping generation algorithm, which maps various classification schemes into GO, and (3) a consensus algorithm to reconcile annotations from the integrated programs and databases.

PIPA's profile generation algorithm is employed to construct the enzyme profile database CatFam, which predicts catalytic functions described by Enzyme Commission (EC) numbers. Validation tests show that CatFam yields average recall and precision larger than 95.0%. CatFam is integrated with PIPA.

We use an association rule mining algorithm to automatically generate mappings between terms of two ontologies from annotated sample proteins. Incorporating the ontologies' hierarchical topology into the algorithm increases the number of generated mappings. In particular, it generates 40.0% additional mappings from the Clusters of Orthologous Groups (COG) to EC numbers and a six-fold increase in mappings from COG to GO terms. The mappings to EC numbers show a very high precision (99.8%) and recall (96.6%), while the mappings to GO terms show moderate precision (80.0%) and low recall (33.0%).

Our consensus algorithm for GO annotation is based on the computation and propagation of likelihood scores associated with GO terms. The test results suggest that, for a given recall, the application of the consensus algorithm yields higher precision than when consensus is not used.

**Conclusion:**

The algorithms implemented in PIPA provide automated genome-wide protein function annotation based on reconciled predictions from multiple resources.

## Background

New sequencing technologies are accumulating proteins with no function annotation at an ever-increasing speed. Traditional experimental methods for determining protein function have proven to be costly and time consuming. Even the use of human curators, who determine protein function from various bioinformatics resources, the literature, and experimental data, will not suffice. Therefore, high-throughput computational tools for accurate and automated protein function prediction are perhaps the only plausible alternative.

Numerous approaches for protein function inference have been proposed [1-4]. These are based on protein homology determined through sequence similarity, structural similarity, function-related sequence and structural features [1], and more sophisticated methods, such as phylogenetic trees [2]. Non-homology-based methods [3], also called genomic context-based predictions, use genomic profiles, gene proximity, and protein interactions for function transfer. The complex relationships between sequence/structure and function lead to errors in function annotation, not only at low sequence identity, where homology is difficult to establish, but also at high sequence identity, where mutations in a few functionally-important sites lead to change in function [4]. However, due to the readily available and fast-growing protein sequence information, sequence homology-based function inference is still the basis for most protein function annotation methods. Compared to direct sequence-based methods, such as function inference through BLAST search, inference based on function-related sequence features, such as domain profiles or motifs, is more accurate and more sensitive for proteins that have low sequence similarity with proteins of known function. This has led to the development and popularity of a wide variety of feature databases, such as Pfam [5], ProDom [6], PROSITE [7], the Clusters of Orthologous Groups (COG) [8], and the Conserved Domains Database (CDD) [9]. Recently, more specialized feature databases have been developed for the prediction of specific protein functions. For example, PRIAM [10] and EFICAz [11] provide profile databases for protein catalytic function predictions. They have proven to be more accurate and sensitive than feature databases developed for general-purpose protein function prediction.

With the existence of many programs and databases that have the capability of inferring different protein functions, a pipeline that properly integrates these resources is able to predict genome-wide protein function with higher accuracy than any individual method. Large integrated information systems, like InterPro [12], BASys [13], GenDB [14], PUMA2 [15], MaGe [16], AGMIAL [17], and IMG [18], are constantly emerging. They include comprehensive resources that allow curators and users alike to gain insights into protein functions. However, these systems are not designed to algorithmically combine different resources for automated protein function prediction. Rather, function information from different resources is usually listed in their original forms, such as accession numbers in a database, and the succinct description of protein functions, reconciling the results from the different resources and eliminating false positive predictions, is edited by human curators. In addition, these systems do not provide tools for database customization to improve the prediction of protein functions of interest.

To address these issues, we describe a new integrated and automated protein function prediction pipeline termed PIPA (Pipeline for Protein Annotation). PIPA differs from other integrated systems as it not only integrates existing programs and databases, but it also allows integration of users' data to predict particular protein functions. This is accomplished through a profile generation procedure for user-categorized protein functions. Most importantly, PIPA combines all integrated resources into a consistent and parsimonious consensus function annotation; a valuable feature that most integrated systems do not provide. The consensus function annotation based on a composite of all resources is potentially able to reduce the effect of false predictions from individual sources, such as databases that are based on protein short motifs, and yield more reliable predictions.

Most established profile databases, such as ProDom and EFICAz, are generated using complex procedures based on either PSI-BLAST [19] or HMMER [20]. The main features of these procedures are the control of profile quality and the generation of multiple profiles for each function related with sequence-divergent proteins. Multiple profiles can be sequentially and iteratively generated from a set of proteins with a common function. This approach has been used to build the ProDom and the PRIAM databases. Conversely, EFICAz builds multiple profiles simultaneously based on clusters of proteins with similar sequences. This reduces the possibility of separating proteins with very similar sequences in the sequential generation of multiple profiles for one function. PIPA adopts the EFICAz procedure. However, unlike EFICAz, it establishes a cut-off threshold for each generated profile. The profile-specific threshold is associated with a user-defined false-positive rate, and it is determined by applying the profile to search a database consisting of functionally related (positive) and unrelated (negative) proteins. The profile-specific threshold assures the accuracy of the functions inferred by the profile. This is an advantage over a profile database with a single threshold, which only assures an average accuracy of the functions inferred by all profiles. We apply the profile generation algorithm to create an enzyme profile database for accurate prediction of protein catalytic functions, named CatFam. CatFam is an integral part of PIPA.

It is challenging for automated computer programs to perform consensus annotation. This is mainly due to the differences in terminology used by various inference methods and the implicit semantic relationships among terms. For example, the fact that one protein is inferred as a "glucokinase" by one method and as a "hexokinase" by another cannot be reconciled unless the computer program knows the relationship between the two terms. In this case, "hexokinase" is a consensus term supported by both predictions, since "glucokinase" is a special type of "hexokinase." The Gene Ontology (GO) consortium [21] has addressed this issue and is dedicated to a consistent description of all gene products. It provides controlled terms and organizes them as a directed acyclic graph.

PIPA adopts GO as a unifying terminology to annotate protein functions. It contains an algorithm to map functions predicted by individual methods using different terminologies (usually database accessions) into GO terms and an algorithm to make consensus predictions based on GO terms.

Mappings from some of the most popular databases to GO terms can be found in the GO website [21]. For databases that do not have mappings for thousands of their families, we use an association rule mining (ARM) algorithm [22] to automatically generate mappings based on samples of proteins with assigned GO terms. The ARM algorithm was previously used to map InterPro identification numbers to Enzyme Commission (EC) numbers [23], where the two databases were considered as two "flat" ontologies. Alternatively, we take into account the hierarchical topology of GO by asserting that: if a GO term can be assigned to a protein, so can its ancestors (i.e., all terms in the path from that term up to the root term of the hierarchy). This helps increase the identification of GO terms for protein families in a database, especially when these families are not related with very specific GO terms that are often used to annotate proteins.

Previously, GO-based consensus was proposed for protein function annotation via multiple matches of GO-annotated protein sequences from a single method, usually BLAST search of a single database [24,25]. The general practice is to propagate the GO terms of matched proteins into a few common ancestral GO terms on the GO hierarchical graph. The ancestral terms are more likely to provide the correct function annotation for the query protein and result in good precision. However, they do not contain as much information (recall) as their descendant terms. One way to achieve a balance between precision and recall is to develop algorithms that assign scores to GO terms and select those terms with scores exceeding a threshold. For example, both GoFigure [24] and GOtcha [25] compute weighted scores for GO terms from the E-value of BLAST hits and propagate them to ancestral terms. GoFigure uses an empirical threshold to select consensus GO terms, while GOtcha infers probability measures for scores of each GO term from background samples. PIPA assigns (heuristically-generated) likelihood scores for GO terms, which indicate the possibility that a GO term is the correct annotation for the query protein. Our algorithm allows users to choose different thresholds for the selection of different consensus terms.

Here, we present the three most important algorithms developed for PIPA: the profile generation procedure, the algorithm for the automated generation of GO mappings, and the GO-based consensus algorithm, which we believe to be the key elements of an integrated and automated protein function annotation system.

## Results and discussion

### Pipeline overview

**Figure 1:**
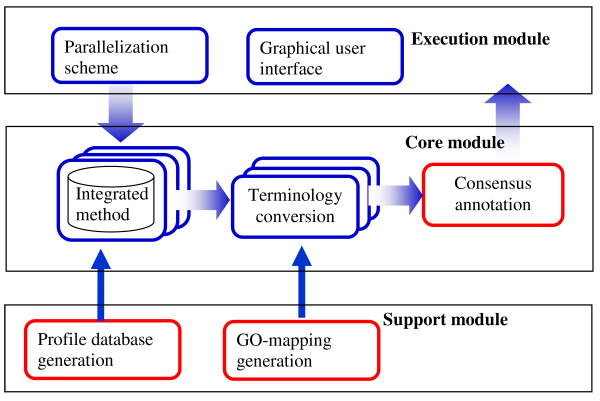
**Overview of PIPA's key modules**. PIPA's programs are organized into three modules. The pipeline execution module consists of programs that enable user access to and control of the pipeline's parallel execution of multiple programs. The execution module wraps the core module, containing all integrated methods (programs and databases), the terminology conversion program, and the consensus annotation program. The support module contains the profile database generation program, which creates new profile databases, and the GO-mapping generation program, which creates GO mappings for the terminology conversion program.

PIPA is designed to allow for easy development of new profile databases and integration of various bioinformatics tools. Figure 1 shows the three modules of the pipeline. The pipeline execution module consists of programs that enable user access to and control of the pipeline's parallel execution of multiple programs. The execution module wraps the core module, containing all integrated methods (programs and databases), the terminology conversion program, and the consensus annotation program. The support module contains the profile database generation program, which creates new profile databases, and the GO-mapping generation program, which creates GO mappings for the terminology conversion program.

Currently, the major integrated methods in PIPA consist of the CatFam database, constructed by the profile database generation program, the 11 publicly-available databases integrated by InterPro, the CDD, the database of Clusters of Orthologous Groups (COG), the transmembrane and signal peptide prediction program Phobius [26], and the bacterial subcellular localization prediction program PSORTb [27]. Table 1 gives a complete list of these resources. PIPA takes as input protein sequences in FASTA format [28] and executes all integrated methods. Parameters, such as E-value cut-off, recommended by the developers of each of the integrated methods are used as the default settings. However, the parameters can be modified to control the rate of false positives in predictions. These predictions, based on their original terminologies, are then converted into GO terms using mapping files. Lastly, the GO consensus annotation algorithm takes these GO terms to determine consensus terms, which are saved, together with the original predictions, in an output file in the General Feature Format [29].

**Table 1:** List of databases/programs in PIPA

Method/Database	Description	Website*
**CatFam**	Enzyme profile databases based on three- and four-digit EC numbers	developed by our group
**CDD**	NCBI Conserved Domains Database	[9]
**COG**	Clusters of Orthologous Groups of proteins	[39]
**Pfam**^+^	Hidden Markov Models of protein domains and families	[40]
**TIGRfam**^+^	Hidden Markov Models of curated protein families	[41]
**SMART**^+^	Identification and annotation of genetically mobile domains	[42]
**Gene3D**^+^	Protein families with structural information	[43]
**FprintScan**^+^	Program that searches the protein fingerprint database PRINTS	[44]
**PANTHER**^+^	Proteins classified by experts into families and subfamilies	[45]
**SUPERFAMILY**^+^	Structural assignments to protein sequences at the superfamily level	[46]
**ProDom**^+^	Automatically generated protein domain families	[47]
**PIR**^+^	Integrated Protein Informatics Resource	[48]
**PROSITE**^+^	Database of protein domains, families and functional sites	[49]
**COILS**^+^	Prediction of coiled-coil regions in proteins	[50]
**Phobius**	A combined transmembrane topology and signal peptide predictor	[51]
**PSORTb**	Prediction of the subcellular localization of bacterial proteins	[52]

*Accessed on July 13, 2007

^+^Integrated from InterPro

PIPA currently integrates 16 programs/databases that are either developed by our group (CatFam) or publicly available.

In the framework of PIPA, CatFam is not only one of its integrated databases that provides catalytic function prediction, but also an example of PIPA's profile generation program, which can be used to generate other specialized databases, provided that sufficient number of sequences is available for clustering and profile generation. Therefore, the evaluation of CatFam's performance in the following section not only demonstrates PIPA's reliability in the prediction of protein catalytic functions but also the effectiveness of its profile generation program.

PIPA is deployed on a LINUX computer cluster at the U.S. Army Research Laboratory's Major Shared Resource Center. All integrated programs are executed in parallel. Using 64 computing processors, PIPA can annotate a typical bacterial genome consisting of 4,000 proteins in about six hours.

### Measures for performance evaluation

There are no universally-accepted approaches to assess the performance of automated function annotation. Here, we use precision and recall, two measures widely-used by the machine learning community, to evaluate the performance of enzyme predictions by CatFam. Precision is the fraction of correctly predicted EC numbers out of all predicted EC numbers, while recall is the fraction of correctly predicted EC numbers out of all EC numbers in the test dataset. In the context of the three-digit EC number prediction, a prediction is considered correct if the first three EC digits match the true EC number.

We evaluate GO predictions by considering the ontology's hierarchical structure in the analysis, so that if one GO term is appropriate to describe a protein function, all of its ancestral terms are appropriate as well. This is also called the true path rule [30]. Therefore, if the prediction of a GO term is its ancestor term, rather than the term itself, the prediction is counted as precise but less specific. In other words, not all information is recalled. Conversely, if a prediction of a GO term is its child term, the prediction is counted as specific but less precise. These considerations led to the extension of the standard definitions of precision and recall, and the establishment of hierarchical precision (HP) and hierarchical recall (HR) for evaluations of GO term predictions [31]. Both HP and HR are normalized to lie in the range [0, 1], and are both equal to one when the predicted annotations completely match the true annotations.

### Enzyme prediction evaluation

We use PIPA's sequence profile generation procedure to construct CatFam. The data used for CatFam development and testing include both enzymes and non-enzymes and are described in the Methods Section. We apply a total of 170,229 proteins for the profile generation. We specify a low false-positive rate of 1.0% (precision 99.0%) to determine the profile-specific cut-off thresholds, and construct databases for three- and four-digit EC number predictions, CatFam-3D and CatFam-4D, respectively. We use a total of 18,949 proteins, not used for profile generation, for CatFam testing. The databases CatFam-3D and CatFam-4D achieve the expected 99.0% precision with 95.5% and 92.5% recall, respectively.

**Figure 2:**
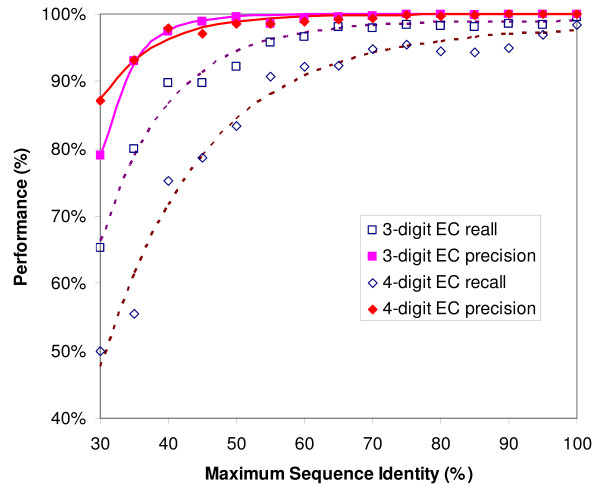
**CatFam performance evaluation**. The performance of CatFam is measured by precision and recall, which are defined as precision = TP/(TP+FP) and recall = TP/(TP+FN), where TP, FP and FN represent the number of true-positive, false-positive, and false-negative predictions, respectively. A total of 18,949 proteins, not used for profile generation, are used to evaluate two CatFam databases, CatFam-3D and CatFam-4D, which predict 3-digit and 4-digit EC numbers for query proteins, respectively. The results are sorted according to the maximum sequence identity between the query protein and the proteins used for profile generation.

To test CatFam's contribution to function prediction for enzymes with low sequence identity to known enzymes, we sort the testing results according to the maximum sequence identity (MSI) between the query protein and the proteins used for profile generation. Figure 2 shows the precision and recall as a function of MSI. All curves have a similar trend; both precision and recall increase with increasing MSI. Both databases achieve more than 90.0% precision and more than 70.0% recall when MSI is decreased to 40.0%.

### Mappings between different ontologies

We develop a procedure that uses the ARM algorithm, detailed in the Methods Section, to automatically generate mappings between two ontologies from sample proteins. We apply this procedure to generate mappings from COG families to GO terms. The sample proteins consist of 31,589 proteins from Swiss-Prot with annotated GO terms. We search these proteins against a COG profile database for matched profiles, determined by a cut-off E-value, that are associated with particular COG families. The ARM algorithm analyzes the COG-GO links and uses two statistics, *support *and *confidence*, to determine a mapping of one COG family to one GO term. *Support *is defined as the number of instances in which a COG family and a GO term appear, and *confidence *represents a conditional probability of the generated mapping. The algorithm accepts a mapping if the associated *support *is greater than 4 and *confidence *is greater than 99.0%.

We apply a similar procedure to generate COG-to-EC mappings, using the sample proteins employed in CatFam generation. These mappings are expected to increase the number of subsequent COG-to-GO mappings through the established EC-to-GO relationship.

**Figure 3:**
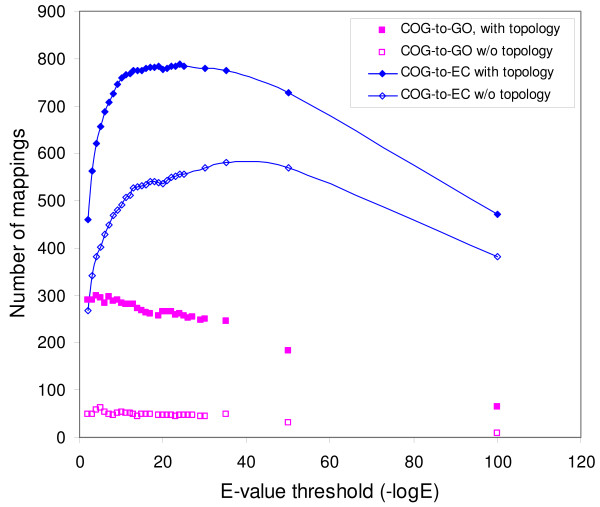
**Mapping evaluation**. The number of automatically generated mappings is significantly increased for properly-selected cut-off E-values when the hierarchical topology of the ontologies is used. Larger cut-off E-values (small values on the *x *axis) result in excessive false hits for sample proteins, while smaller cut-off E-values exclude true hits. Both cases reduce the number of mappings that can be generated.

Figure 3 shows a parametric study of the number of generated mappings as a function of cut-off E-values. Although typical cut-off E-values, such as 10^-2^, have been used in some applications [32], we try a series of cut-off values to find the best operating point at which the maximum number of mappings might be found. A trend can be observed for COG-to-EC mappings, where the number of generated mappings increases to a maximum and then decreases as the cut-off E-values decrease. The increase is explained by the removal of false-positive matches with the reduction in the cut-off values. The decrease after the maximum is due to the elimination of true-positive matches when the cut-off value is further reduced. The smaller number of mappings generated for larger cut-off E-values also suggests that the mapping generation process can avoid misleading false-positive matches. More importantly, Figure 3 shows that considering the hierarchical structures in the GO and the EC ontologies help generate significantly larger number of mappings. In particular, six times more COG-to-GO mappings and over 40.0% more COG-to-EC mappings are generated with a properly selected E-value threshold.

In order to evaluate the accuracy of the automatically-generated mappings, we perform a 20-fold cross-validation, where we randomly select 95.0% of the proteins from the 31,589 sample proteins to generate the mappings and use the remaining 5.0% for testing. We repeat this procedure 20 times, each with different partitioning of the dataset. Table 2 shows the result. The COG-to-GO mappings achieve moderate average precision (80.0%) and low average recall (33.0%) for GO annotation. However, the COG-to-EC mappings, evaluated in a similar cross-validation procedure, achieve excellent performance with 99.8% precision and 96.6% recall.

**Table 2:** Mapping evaluation (cross-validation)

Ontology Mapping		Hierarchical Precision	Hierarchical Recall
COG-to-GO	Function	77.6%	24.3%
	Process	75.5%	20.0%
	Component	86.0%	54.8%
	**Average**	**79.7%**	**33.0%**
COG-to-EC		**99.8%**	**96.6%**

The accuracy of the automatically-generated mappings is evaluated by a 20-fold cross-validation process using 31,589 sample proteins. The COG-to-GO mappings achieve moderate average precision (80.0%) and low average recall (33.0%) for GO annotation. However, the COG-to-EC mappings, evaluated in a similar cross-validation procedure, achieve excellent performance with 99.8% precision and 96.6% recall.

The comparison of COG-to-GO and COG-to-EC mappings indicates that the number and quality of the automated mappings strongly depend on the annotation accuracy and completeness of the sample proteins used for mapping generation. For example, if a GO term is assigned to only half of the proteins that should have that GO annotation and all of these proteins match one COG family, the observed confidence for the mapping of this COG family to the GO term would be only 50.0%, and this mapping would be discarded. Actually, we find cases in which the correct GO terms are not assigned to proteins, especially for enzyme annotations in the Swiss-Prot database. The absence of GO terms could explain the fact that the number of automatically-generated COG-to-GO mappings is much smaller than the number of COG-to-EC mappings generated in a similar way (Figure 3). More GO mappings are expected to be generated with the addition of new curated GO annotations to the Swiss-Prot database.

### Evaluation of GO-based consensus annotations

We determine consensus GO terms for protein predictions from distinct individual sources by considering the mapped GO terms and their ancestral terms. Initially, our algorithm assigns scores to each GO term for each individual source that infers that GO term. For consistent scoring across the different prediction algorithms, each individual score is calculated based on the E-value of the prediction and is scaled between zero and one using the corresponding cut-off E-values *E*_0 _and *E*_1_, respectively, as explained in the Methods Section. A minimum score of zero is assigned to a prediction if the corresponding E-value is equal to or greater than *E*_0_, and a maximum score of one is assigned if the E-value is equal to or smaller than *E*_1_. Each GO term acquires a final score based on all of its individual scores and composite scores propagated to it from its descendants through the GO topology. The terms with final scores greater than a pre-selected score acceptance threshold (SAT) are included in the consensus prediction.

**Figure 4:**
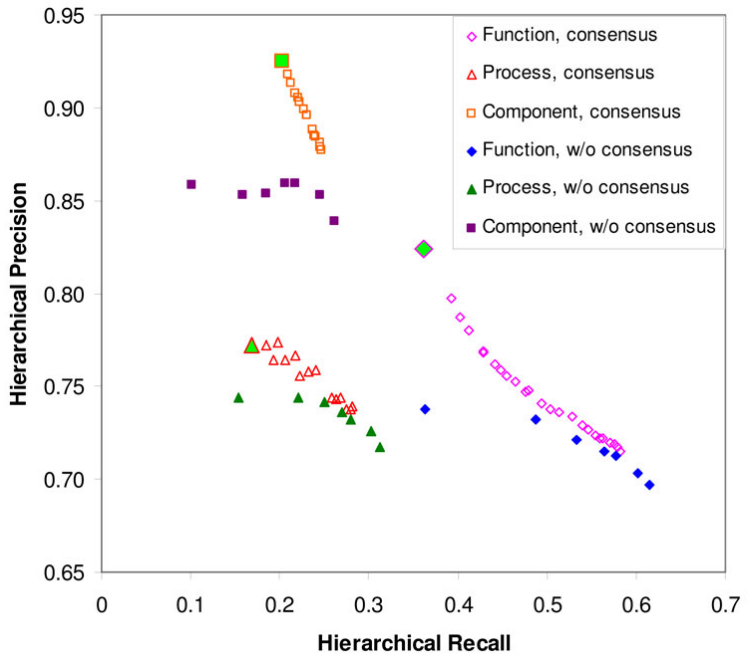
**GO consensus evaluation**. PIPA's GO annotations with and without the consensus algorithm are compared for hierarchical precision (HP) and hierarchical recall (HR). For the consensus algorithm, each data point in the figure is computed by using different combinations of the three parameters, *E*_0_, *E*_1_, and SAT. When the consensus algorithm is not used, each data point is obtained by selecting a different cut-off E-value. The figure indicates that the consensus algorithm improves HP for each of the three GO categories. The highlighted points correspond to consensus algorithm results with parameters *E*_0 _= 0.01, *E*_1 _= 1e-200, and SAT = 0.99.

We employ the 31,589 proteins from Swiss-Prot with annotated GO terms to test the consensus algorithm. The hierarchical precision HP provides a measure of the accuracy of the GO-term predictions, and hierarchical recall HR provides a measure of the coverage of the predictions. Figure 4 compares the performance of GO annotations with and without the consensus algorithm for all three GO categories: molecular function, biological process, and cellular component. Data points corresponding to the consensus algorithm are obtained by changing parameters *E*_0_, *E*_1_, and SAT. Without consensus, adjusting the performance of GO annotations is achieved by changing the cut-off E-value. The figure suggests a significant trade-off between precision and recall. However, for a given recall, the application of the consensus algorithm yields higher precision than when consensus is not used. Precision is improved by up to 8.0% for both molecular function and cellular component, and by up to 4.0% for biological process. The highest precision is achieved for the parameters set as *E*_0 _= 0.01, *E*_1 _= 10^-200^, and SAT = 0.99 for all three GO categories, which are highlighted in the figure with larger-size markers.

The results suggest that the consensus algorithm effectively integrates different function inferences to improve the precision of GO annotation. The low HR, which indicates a low coverage of GO terms predicted by the pipeline, is likely due to the incompleteness of the GO mappings that link individual databases with GO terms and the limited coverage of the integrated databases for the prediction of biological processes and cellular components. The existing mappings to GO terms from PIPA's two major sources, InterPro and CatFam, cover a total of 4,379 molecular function terms, 1,053 biological process terms, and 266 cellular component terms, whereas the 31,589 testing proteins contain 2,814 molecular function terms, 4,517 biological process terms, and 907 cellular component terms. This means that no more than 23.0% of the biological process terms and 29.0% of the cellular component terms associated with the testing proteins can be covered by existing GO mappings. We expect that the addition of GO mappings for existing databases and the integration of new methods, capable of predicting currently underrepresented protein functions, will increase the coverage of GO annotations in PIPA.

### Current limitations and plans for improvement

Perhaps, one of PIPA's main limitations is that all of its currently integrated resources to predict protein function use annotation transfer based on sequence homology. Sequence homology is the most established approach for protein function prediction. However, we are planning on expending PIPA's function prediction capabilities by incorporating comparative analysis approaches, e.g., phylogenetic tree analysis, to prevent function transfer errors caused by gene duplication or gene loss. Future work may also include the incorporation of non-homology-based prediction methods. For example, *de novo *function prediction based on machine-learning algorithms with sequence-derived features seems very promising [33]. They may provide valuable resources for predicting orphan proteins, which do not have significant sequence similarity with known proteins. However, such methods require substantial training data and are thus limited to well-populated Gene Ontology categories. Function prediction methods based on protein-protein interaction networks [34] are also becoming important due to the increased availability of protein interaction data. However, experimental uncertainty of protein interaction data makes these methods unsuitable for automated, large-scale implementation, at this stage of development.

Another perceived limitation of PIPA relates to the potential false-positive predictions inferred by integrated methods that are based on short motifs. In its current configuration, PIPA has some mechanisms to alleviate this problem. First, for methods that are dependent on short motifs and systematically yield excessive false positives, PIPA can reduce them by restricting the E-value cut-offs. Second, because PIPA's function annotation is based on the consensus of different integrated approaches, most of which are not based on short motifs, the effect of false-positive predictions from individual methods is mitigated. However, the consensus algorithm cannot completely eliminate false predictions introduced by short motifs prevalent in different function domain databases. In future developments, we will explore alternative solutions based on data mining [35], which are similar in spirit to the approaches that we have already applied to generate mappings between ontologies.

## Conclusion

We have developed methods for an integrated and automated protein function annotation pipeline. The three main algorithms presented here improve annotation accuracy by providing the means to develop customized profile databases and by exploiting and consistently consolidating protein function information from disparate sources based on different terminologies. An added benefit is that the consolidated function predictions are given in GO terms, which is becoming the *de facto *standard in the community.

We show the effectiveness of the profile generation procedure for particular protein functions through the development of CatFam, which not only achieves overall excellent precision and recall but also performs well for enzymes with low sequence identity. The clustering procedure and the use of negative samples have contributed to the quality of the generated profiles. In addition, the use of profile-specific thresholds ensures equal accuracy for each profile and avoids the problem of having a single E-value threshold for all profiles, which yields good overall results but poor performance for some profiles. Moreover, the introduction of negative samples allows users to set a false-positive rate for the resulting database.

Although PIPA achieves very good performance for catalytic function annotation with the CatFam databases, its overall performance for other categorical functions is dependent on the various integrated resources. PIPA's profile generation algorithm may be helpful in developing methods to annotate some of these functions, however, for other functions, such as protein subcellular location inferred with PSORTb and transmembrane proteins inferred with Phobius, highly specialized methods are irreplaceable.

We adopt GO as the unifying protein annotation terminology to fuse various functions inferred from different sources. We demonstrate that mappings between terminologies used by different sources and GO can be generated by the ARM algorithm from samples of annotated proteins. The significantly increased number of identified mappings suggests that GO's hierarchical topology must be considered during the mapping generation. It provides the opportunity to link a broad functional category in a database with a generic GO term that is infrequently used to annotate proteins.

Concise and more accurate GO annotations can be obtained by the proposed consensus algorithm. The ability to optimize the algorithm's parameters and the future availability of additional reliable GO mappings will further improve consensus predictions.

It should be noted that PIPA is more than a readily available comprehensive protein function annotation pipeline. It is an open framework for incorporating different function prediction methods, homology-based or non-homology-based, whenever they become mature and available. As additional computational methods are incorporated, PIPA will expand the functional categories of annotated proteins. This will improve annotation reliability through the consensus procedure, which mitigates potential false predictions from individual methods. In addition, PIPA's modular parallelization framework will maintain the pipeline's high-throughput capability after integration of any number of resources.

## Methods

### Data preparation

All data used in this paper are from the Swiss-Prot database (UniProtKB/Swiss-Prot 51.1) and from the Enzyme Nomenclature Database (END), both released on November 14, 2006. These consist of 75,687 enzymes annotated by END and the corresponding sequences from Swiss-Prot. Of these, a randomly selected set of 68,087 (90.0%) are used for generating (training) CatFam and the remaining 7,600 for testing. In addition, we use a total of 113,491 non-enzyme proteins from Swiss-Prot as negative examples, where 90.0% are used for training CatFam and the remaining 10.0% for testing. Hence, the entire training and testing data sets consist of 170,229 and 18,949 proteins, respectively.

We employ a total of 31,589 proteins with annotated GO terms from Swiss-Prot for generating mappings between different ontologies and GO and evaluating the GO consensus algorithm. This set only includes reliable GO annotations and, therefore, excludes annotations with evidence codes IEA (Inferred by Electronic Annotation), NAS (Non-traceable Author Statement), and ND (No biological Data available). Among the 120,783 GO annotations for these proteins, 21,418 (17.7 %) are labeled with ISS (Inferred from Sequence or Structural Similarity) evidence codes. We consider these annotations as reliable because, according to the guide to GO evidence codes [36], ISS is part of the "Curator-assigned Evidence Codes," where human curators have reviewed the annotations initially inferred from sequence or structural similarity.

### Sequence profile database generation

A sequence profile generated from protein sequences of a common function reveals the functionally conserved amino acid patterns on the sequences. Hence, a protein that matches such a profile will be annotated by the function associated with the profile. We construct a database of sequence profiles from proteins with known functions. The proteins are grouped by their different functions, where each group forms a training set for a specific function. One or multiple profiles are generated for each function. In addition, a separate testing database, consisting of all of these proteins and other proteins with irrelevant functions (negative examples), is used to test a profile's performance during the profile generation procedure. The following steps describe this procedure:

1. For a given protein function, estimate pair-wise sequence similarity for proteins in the training set associated with that function. This is achieved through an all-against-all PSI-BLAST search, where E-values are used as the similarity score.

2. Based on sequence similarity (E-values), employ a hierarchical clustering algorithm [37] to group proteins of the given function into distinct clusters. Initially, each sequence forms a cluster. Then, perform a pair-wise search among all clusters and merge two clusters, *C*_*i *_and *C*_*j*_, that have the smallest cost function

(1) *F*(*C*_*i*_, *C*_*j*_) = *max*[*E*(*a*,*b*), ∀*a *∈ *C*_*i*_, ∀*b *∈ *C*_*j*_]

into one cluster. Here, *E*(*a*,*b*) denotes the E-value between protein sequences *a *and *b *in clusters *C*_*i *_and *C*_*j*_, respectively. Sequentially continue this merging procedure until the cost function *F *exceeds a specified limit.

3. Generate one profile for each cluster. A profile generation begins by performing multiple sequence alignments (MSA) with ClustalW [38] for a subset of the most similar protein sequences in the cluster. Record the number of conserved positions in the MSA.

4. The MSA is provided as input to PSI-BLAST, which generates a profile in the format of a position specific scoring matrix (PSSM). Next, search for proteins that match the profile in the testing database, consisting of proteins of all functions. Taking a raw score as a cut-off value, find protein matches of the same function as the profile (true-positive hits) and some protein matches to other functions (false-positive hits). Determine the lowest raw score cut-off for the profile, termed raw score threshold (RST), so that the false-positive rate for matched proteins is smaller than a specified value.

5. Add one additional protein to the MSA in Step 3 and repeat Step 4. Based on the pair-wise sequence similarity computed in Step 1, the newly added protein has the most similar sequence with those in the MSA. Continue this iteration until the number of conserved positions, i.e., columns of identical amino acids, in the MSA is reduced to one.

6. Compare all PSSM profiles created in Step 4, and select the one with the maximum number of true positive hits as the final profile for that cluster.

7. Repeat Steps 3–6 for all clusters generated in Step 2.

8. Repeat Steps 1–7 for all protein functions. The profiles for all functions are stored with their corresponding RSTs in a RPS-BLAST searchable database.

This procedure is used to generate the enzyme profile database CatFam for both three- and four-digit EC numbers, CatFam-3D and CatFam-4D, respectively. Because the profiles generated in Step 4 above use a database containing both positive and negative samples, each database can be generated with a specified false-positive rate and each profile is associated with a specific threshold (i.e., RST). This is a distinct feature of CatFam, which, in a sense, allows developers to guarantee a false-positive rate of the predictions for each function.

### Algorithm for mappings among different ontologies

Mapping among different ontologies is the process of translating terms from one ontology into another. To automate this process, we first link terms between two ontologies by applying terms of one ontology to re-annotate a set of sample proteins that have been annotated with another ontology. Next, we apply the ARM algorithm [21] to extract the underlying mappings between the two ontologies from the sample proteins. Following, we describe the algorithm to generate the mappings from COG families to GO terms, given a set of GO-annotated sample proteins **D**, consisting of the 31,589 proteins from Swiss-Prot database described above.

1. For one sample protein in set **D**, with known GO terms denoted as *G*, apply RPS-BLAST to search the COG profile database for matches bellow a given cut-off E-value. The resulting COG-family IDs, denoted as *C*, and GO terms *G *form one instance that links COG and GO terms, denoted as I(*C*→*G*).

2. Extend the set *G *in I(*C*→*G*) by including all ancestral GO terms associated with *G*.

3. Repeat Steps 1 and 2 for all sample proteins in **D**.

4. For all instances of links I(*C*→*G*), obtain the "true" mappings between COG family IDs and GO terms. The ARM algorithm searches for pairs of COG ID and GO term, denoted as (*c*, *g*), and for each pair calculates two statistics: *support*(*c*, *g*) and *confidence*(*c*→*g*), which are defined as

(2) *support*(*c*, *g*) = the number of instances that contain both terms *c *and g

(3) *confidence*(*c*→*g*) = *support *(*c*, *g*)/*support *(*c*)

5. If both *support*(*c*, *g*) and *confidence*(*c*→*g*) exceed a specified threshold, the mapping from COG ID *c *to GO term *g *is generated. However, this mapping from *c *to *g *is not considered if *g *is an ancestor of some GO term *g' *whose *c *to *g' *mapping has already been generated.

A similar procedure is applied to generate COG to EC mappings employing the 170,229 EC-annotated proteins used to construct CatFam, as discussed above.

### Hierarchical consensus prediction

We develop a consensus algorithm to reconcile GO-term annotations for a query protein inferred from multiple sources, such that the reconciled (consensus) GO annotations are more accurate yet specific enough for the description of the protein's functions. The algorithm is based on heuristically-generated likelihood scores, ranging from 0 to 1, which indicate the likelihood that a GO term is the correct annotation for the query protein. The higher the score is, the more likely the GO term is the correct annotation. The following steps detail the consensus algorithm.

1. For a given query protein, identify a set *F *of GO terms *f*, where *f *∈ *F*, and sets of E-values *E*_*f*_, where each *e*, with *e *∈ *E*_*f*_, is the E-value from one individual source that infers GO term *f*. Each GO term *f *is assigned one evidence score *l*(*f*,*e*) from each source with associated E-values *e*, given by the following equation

(4) $$l(f,e)=\{\begin{array}{ll} 0, & e\geq E_{0}\\ \frac{\log(e/E_{0})}{\log(E_{1}/E_{0})}, & E_{1}<e<E_{0}\\ 1, & e\leq E_{1} \end{array}$$

where *E*_1 _and *E*_0 _correspond to preset upper and lower bounds on E-values, respectively. The evidence score is set to 0 for E-values equal to or larger than *E*_0_, and to 1 for E-values equal to or smaller than *E*_1_. Different sources may use different values for *E*_1 _and *E*_0_.

2. When different sources happen to infer the same GO term *f*, compute a composite evidence score *L*_*S*_(*f*) for that term,

(5) $$L_{S}(f)=1-\prod_{e\in E_{f}}\left(1-l(f,e)\right).$$

3. Next, propagate the composite evidence score upwards for all ancestors of *f*. For a given ancestor *q *of GO-term *f*, the propagated evidence score *L*_*P*_(*f,q*) is given as

(6) LP(f,q)={LS(f)+(1−L(f)S)×NqN0,LS(f)>00,LS(f)=0

where *N*_*q *_and *N*_0 _are the number of leaf terms under the *q *term and the root term, respectively. Note that equation (5) assigns 1 to *L*_*P *_for the root term if any of its descendants has a non-zero composite evidence score. This allows for consistent assignment of maximum likelihood values to each of three root GO terms, molecular function, biological process and cellular component, which can always be assigned to a given protein.

4. Finally, each GO-term *q *gets one final score *L*(*q*),

(7) $$L(q)=1-(1-L_{S}(q))\times \prod_{f\in C_{q}}\left(1-L_{P}(f,q)\right)$$

where *C*_*q *_is the set of GO terms that are descendants of *q *in set *F*.

5. The consensus GO terms for the query protein are identified by scanning all GO terms and selecting the ones that have a final score *L *greater than a specified score acceptance threshold. If a GO term and one of its ancestors are both selected, the ancestor annotation is eliminated from the consensus, yielding a more specific set of annotations.

## Authors' contributions

All authors contributed to the development of the methodology. CY and NZ led method conceptualization and prepared the original draft, which was revised by JR. CY and VD performed most implementations. All authors read and approved the final manuscript.
